# Unwinding and Rewinding: Double Faces of Helicase?

**DOI:** 10.1155/2012/140601

**Published:** 2012-07-19

**Authors:** Yuliang Wu

**Affiliations:** Department of Biochemistry, University of Saskatchewan, Health Sciences Building, Saskatoon, SK, Canada S7N 5E5

## Abstract

Helicases are enzymes that use ATP-driven motor force to unwind double-stranded DNA or RNA. Recently, increasing evidence demonstrates that some helicases also possess rewinding activity—in other words, they can anneal two complementary single-stranded nucleic acids. All five members of the human RecQ helicase family, helicase PIF1, mitochondrial helicase TWINKLE, and helicase/nuclease Dna2 have been shown to possess strand-annealing activity. Moreover, two recently identified helicases—HARP and AH2 have only ATP-dependent rewinding activity. These findings not only enhance our understanding of helicase enzymes but also establish the presence of a new type of protein: annealing helicases. This paper discusses what is known about these helicases, focusing on their biochemical activity to zip and unzip double-stranded DNA and/or RNA, their possible regulation mechanisms, and biological functions.

## 1. Introduction

Helicases are molecular motors that couple the energy of nucleoside triphosphate hydrolysis to the unwinding and remodeling of structured DNA or RNA [1–3]. The number of helicases expressed in higher organisms is strikingly high, with approximately 1% of the genes in many eukaryotic genomes apparently encoding RNA or DNA helicases. Helicases are involved in virtually all aspects of nucleic acid metabolism, including replication, repair, recombination, transcription, chromosome segregation, and telomere maintenance [4–7]. Based on their substrates, helicases can be classified as DNA or RNA helicases, although some can function on both DNA and RNA molecules [8]. DNA helicases have been reported to function in a variety of DNA metabolic processes, including unwinding duplex or alternative DNA structures (triplex, G-quadruplex), stripping protein bound to DNA, and chromatin remodeling [5, 6, 9, 10].

**Figure 1:**
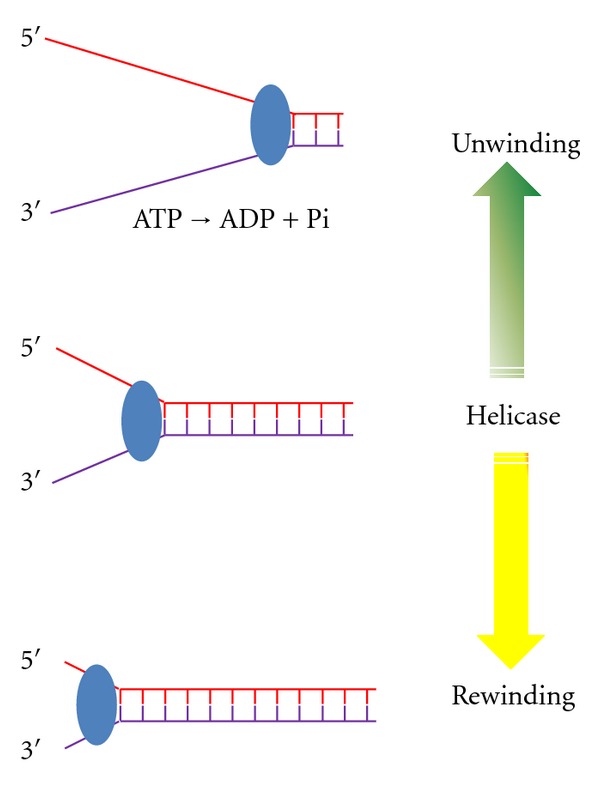
A model of helicase function to unwind or rewind double-stranded nucleic acid. Note that helicase (oval) represents both translocation polarities; either ATP-dependent or ATP-independent for strand annealing activity has been observed in helicases (see Table 1).

Traditionally, helicases are known to unwind double-stranded DNA or RNA in an ATP-dependent manner. However, increasing evidence suggests that some helicases can rewind, or anneal, complementary strands of polynucleic acids in the presence or absence of nucleoside triphosphate (Figure 1). Moreover, two so-called human helicases that were identified recently appear to only have ATP-dependent rewinding activity [11–15]. These discoveries not only enrich the definition of helicases but also establish the presence of a new type of protein: annealing helicase. The mechanism of this novel strand annealing activity and its biological consequences remain largely unknown. In this paper, I will provide a brief overview of strand annealing activity found in various proteins across species and then focus on annealing helicases in humans and their potential mechanisms and functions.

## 2. Proteins with Strand Annealing Activity

Most knowledge regarding the strand annealing activity of proteins has come from studies of model systems, including bacteria, yeast, and *Xenopus laevis*. The Lehman lab reported the ATP-dependent annealing activity of purified recombinant *Escherichia coli* RecA protein three decades ago [16]. Although similar results were obtained by several other labs [17–19], the annealing activity of RecA (the human RAD51 homolog) is likely due to the binding of single-stranded DNA (ssDNA), which forms a nucleoprotein filament. The RecA (RAD51) searches for homology along double-stranded DNA (dsDNA) and forms a stretching of DNA duplex. Two other bacterial proteins—regulator protein StpA and recombination mediator protein RecO—have been reported to promote annealing of complementary single stranded RNA (ssRNA) and ssDNA, respectively [20, 21]. The DNA replication polymerase (Dpo1) of *Sulfolobus solfataricus* also possesses strand annealing activity [22]. In yeast, the Rothstein lab first found that Rad52 protein binds ssDNA and dsDNA and promotes strand annealing [23]. Later, the Kowalczykowski lab demonstrated that Rad51 and Replication Protein A (RPA) regulate Rad52's annealing function [24–26]. Another yeast protein, Rad59, also stimulates complementary ssDNA annealing [27, 28]. *Xenopus* RNA-binding protein X1rbpa was reported to promote RNA : RNA annealing [29]. Interestingly, strand annealing activity has also been reported for some viral proteins. For example, Tat, one of six HIV regulatory proteins, encodes a small nuclear transcriptional activator and stimulates DNA : DNA annealing [30, 31]; dengue virus core protein also promotes RNA : RNA annealing [32].

Several mammalian proteins have been found to possess annealing activity. The Wilson lab first reported that rat heterogeneous nuclear ribonucleoprotein (hnRNP) A1 has strand annealing activity on both DNA and RNA [33], and the activity is inhibited by phosphorylation of kinase PKA or PKC. The annealing activity is restored by dephosphorylation of hnRNP by phosphatase 2A [34, 35]. Mouse p53 protein inhibits the unwinding activity of T antigen helicase *in vitro* [36]; human p53 protein was also later found to inhibit several other helicases' unwinding activity due to its strong DNA : DNA and RNA : RNA annealing activity [37]. Human Mre11 complex mediates the annealing of complementary ssDNA molecules [38]. The human RAD51B, RAD51C, RAD51D, and XRCC2 protein complex catalyzes the strand-annealing reaction between a long linear ssDNA (1.2 kb in length) and its complementary circular ssDNA [39]. In summary, at least a dozen proteins, particularly those involved in DNA replication and repair, have been demonstrated to possess strand annealing activity. However, it is surprising to find that helicases, which unwind double stranded DNA or RNA, also possess strand annealing activity.

## 3. The Discovery of the Annealing Activity of Helicases

The annealing activity was first reported in RNA helicases (Table 1). In 1997, the Busch lab reported that human RH II/Gu RNA helicase has RNA folding activity—forming an intramolecular duplex [40]. In 2001, the Stahl lab discovered that RNA helicase p68 and its close relative p72 possess RNA annealing activity for two complementary RNA strands [41], and they also showed that Ddx42p, another p68 homolog, has similar activity [42]. RNA helicase A, also known as nuclear DNA helicase II or DHX9, is a superfamily 2 (SF2) helicase that unwinds DNA-DNA, RNA-RNA, and DNA-RNA strands with a 3′-5′ polarity [43]. Recently, it was reported that RNA helicase A promotes the annealing of tRNA_3_^Lys^, the primer for reversing transcription, to HIV-1 RNA [44]. In addition to human RNA helicases, some yeast and bacterial RNA helicases also contain annealing ability. For example, the Jankowsky lab demonstrated that DED1 from *Saccharomyces cerevisiae*, in addition to its RNA unwinding activity, facilitates the formation of RNA duplexes [45]. The Lambowitz lab found that Mss116p of *S. cerevisiae* promotes the annealing of the oligonucleotides in the absence of ATP [46]. CrhR, a cyanobacterial RNA helicase, was also found to promote duplex formation in the presence of ATP [47]. Dengue virus RNA helicase NS3 accelerates RNA annealing in the absence of ATP [48].

**Figure 2:**
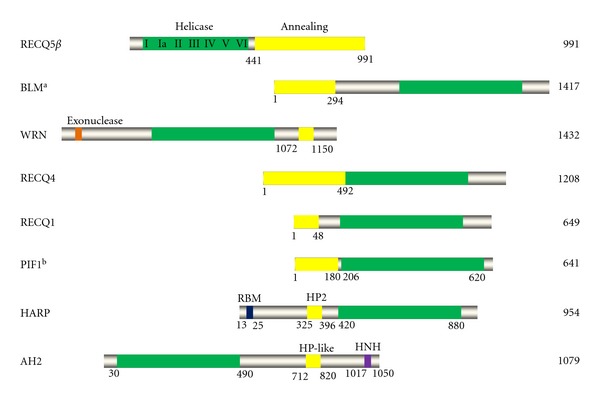
Alignment of human DNA helicases that contain annealing activity. The annealing domain is indicated in yellow and the conserved helicase core domain in green; the conserved seven helicase motifs and the accessory domains are indicated. HP2, HepA-related protein domain 2; RBM, RPA-binding motif; HNH refers to the three conserved His and Asn amino acid residues in the motif. The total number of amino acids in each helicase protein is shown to the right. (a) It was reported that the C-terminus of BLM is important for its strand-annealing function [51, 53]; (b) it was also reported that the helicase core domain of PIF1 has annealing activity [66]. The functional annealing activity domain for CSB, TWINKLE, and Dna2 has not been mapped.

Compared with RNA helicase, more DNA helicases have been found to possess annealing activity (Table 1). The Janscak lab is the first to report that both unwinding and annealing activity resided in a DNA helicase, RECQ5*β* in 2004 [55]. RECQ5*β* is the longer isoform of RECQ5 helicase and is localized in the nucleus. The Janscak lab not only discovered the novel activity of RECQ5*β*, but also mapped the unique C-terminal portion (aa 411–991) that possesses the DNA strand-annealing activity [55]. This was the first demonstration of a DNA helicase with an intrinsic DNA annealing function residing in a separate domain of the same polypeptide (Figure 2). Subsequently, many helicases, particularly RecQ family helicases, have been found to possess annealing activity. In humans, there are five RecQ homologs, and mutations in three of these genes (*BLM*, *WRN*, and *RECQ4*) are associated with Bloom's, Werner, and Rothmund-Thomson syndromes, respectively. Although no disease has been linked to mutations of RECQ5, recq5^−/−^ mice are highly cancer prone and display genomic instability [60, 61]. A single nucleotide polymorphism in RECQ1 correlates with decreased survival of pancreatic cancer patients [62, 63]. Annealing activity was reported for the Bloom's syndrome helicase, BLM, in 2005 by two independent groups [51, 53]. Recently, the Brill lab further identified a subdomain of the BLM/Sgs1 N-terminus that contains annealing activity *in vitro* [64]. Not only do human BLM and yeast Sgs1 display annealing activity, so does the *Drosophila *BLM homolog (mus309/DmBLM) [51, 64, 65]. The WRN helicase was reported to contain strand pairing activity [51], and the annealing activity was mapped to the C-terminal region (aa 1072–1150) [52]. Annealing activity was also found for human RECQ1 protein by the Brosh lab [49]. Later, the Vindigni lab reported that RECQ1 efficiently promotes strand annealing as a higher order oligomer (pentamer or hexamer), while smaller oligomeric states (dimer or monomer) act to unwind duplex DNA [50]. Different quaternary states of RECQ1, modulated by binding of ssDNA and ATP, are associated with its strand annealing or unwinding activity (discussed later).

Unlike other RecQ helicases, neither endogenous RECQ4 purified from HeLa cell extracts [67] nor recombinant RECQ4 purified from *E. coli* [68] originally showed unwinding activity. The unwinding activity of RECQ4 appears to have been masked by its strong intrinsic DNA annealing activity [54, 69]. Because of its robust annealing activity, the presence of a third strand (e.g., same sequence as the labelled oligo but unlabelled) is required to demonstrate unwinding by RECQ4. Furthermore, the N-terminus (aa 1–492) is required for the annealing activity of RECQ4 [54]. Although it was later reported that RECQ4 could displace short oligonucleotides (≤22 mer) without a third strand, the fact that longer oligonucleotides (≥30 mer) require a third strand [69, 70] indicates the strong annealing activity resides in the RECQ4 peptide.

In addition to the RecQ helicase family, several other DNA helicases possess strand annealing activity too (Table 1). The Pif1 helicase family is a group of 5′ → 3′ directed, ATP-dependent, super-family IB helicase that is found in nearly all eukaryotes [71]. The purified recombinant human PIF1 proteins display robust annealing activity without ATP, and this activity resides in the N-terminal region (aa 1–180) of the protein [56] (Figure 2). Dna2 is a helicase and nuclease involved in Okazaki fragment processing, double-strand break (DSB) repair, telomere regulation, and mitochondrial function [72]. Both yeast and human DNA2 protein contain strand annealing properties. Point mutation analysis revealed that the annealing activity does not require either the nuclease or the helicase activity of Dna2 [57], indicating that the rewinding activity is uncoupled from nuclease and/or unwinding activity. Mutations in *CSB* gene cause Cockayne syndrome, a rare inherited genetic disorder characterized by UV sensitivity, severe neurological abnormalities, and progeroid symptoms [73]. The CSB protein catalyzes strand annealing of complementary ssDNA, while both ATP and RPA inhibit its annealing activity [58]. TWINKLE is a human mitochondrial DNA helicase that is associated with heritable neuromuscular diseases, and it has NTP-independent annealing activity [59]. The T4 phage UvsW helicase [74] two archaea helicases (Hjm/Hel308A [75] and Hel112 [76]), and *Mycobacterium tuberculosis* XPB [77] also contain strand annealing activities.

## 4. Annealing Helicases HARP and AH2

The term “annealing helicase” was created when two helicase domain-containing proteins were discovered to possess annealing activity and no unwinding activity. In 2008, the Kadonaga lab discovered that HARP is an ATP-dependent annealing helicase [14]. HARP (HepA-related protein), also known as SMARCAL1 (SWI/SNF-related, matrix-associated, actin-dependent regulator of chromatin, subfamily a-like 1), is a member of the SWI/SNF family protein. Mutations in HARP are associated with Schimke Immuno-Osseous Dysplasia (SIOD), a multisystem autosomal recessive disorder characterized by short stature, kidney disease, and a weakened immune system [78]. The majority of SIOD patients have T-cell deficiency and associated risk for opportunistic infection, a common cause of death. Loss of HARP affects cellular proliferation and differentiation, and the response to replication stress [79]. Following the Kadonaga lab's report, three other labs found similar enzymatic activity for HARP and further defined its biological functions [11–13]. HARP binds directly to RPA via a conserved N-terminal motif and anneals RPA-coated complementary ssDNA. It was proposed that HARP might dictate its role in the S-phase-specific DNA damage response to protect stalled replication forks by minimizing the accumulation of ssDNA regions and facilitating the repair of collapsed replication forks [11–13, 80]. Similar results were also obtained for HARP in a *X. laevis* system [81]. The Chen lab further identified that two HP domains, each has about 60 residues, dictate the annealing activity of HARP [82]. More recently, the Cortez lab reported that the first HP domain is not required for annealing activity, and, intriguingly, HARP is able to catalyze branch migration of Holliday junctions (HJs) and regression of replication forks [83]. 

After discovering the unique activity of the HARP protein, the Kadonaga lab identified another annealing helicase that they named annealing helicase 2 (AH2) [15]. AH2 was previously named ZRANB3 (zinc-finger, RAN-binding domain containing 3), a member of the Snf2 family [84]. Similar to HARP, the purified recombinant AH2 protein displays DNA-dependent ATPase activity and ATP-dependent rewinding activity. However, unlike HARP, AH2 lacks a conserved RPA-binding domain and does not interact with RPA. In addition, AH2 contains an HNH motif at its extreme C-terminus (Figure 2), which is common in prokaryotes and is often associated with nuclease activity [85]. Contrary to expectations, the purified recombinant AH2 protein does not exhibit nuclease activity [15]. With no disease linked to AH2 or genetic model generated for AH2, the biological function of AH2 remains largely unknown.

Interestingly, the direction of both annealing helicases (HARP and AH2) has not been examined *in vitro*. Since classic DNA-dependent ATPases that are bonafide helicases that have the ability to catalytically separate complementary strands behave in a directional manner with respect to the strand that the helicase protein is presumed to be bound, depend on which we define 3′-5′ or 5′-3′ helicase, therefore, it would be of interest to know whether HARP or AH2 translocate in a directionally specific manner.

## 5. Possible Mechanisms of Helicase Annealing Activity 

Specific helicases need to function on the appropriate nucleic acid substrate at the appropriate time. In addition, these enzymes might be required to facilitate unwinding and/or rewinding under different circumstances. Indeed, several Bloom's syndrome missense mutant proteins lack unwinding activity, but still possess strand annealing activity that is even greater than wild type BLM [86], indicating that the misregulation of unwinding and rewinding activity may be one of the pathogenic factors. A key emerging question is how these two opposite activities of helicase are precisely regulated.

### 5.1. Do Helicases Contain an Annealing Domain?

Helicase is characterized by conserved helicase motifs. The number of helicase motifs varies from seven, nine, to twelve, which are responsible for nucleic acid binding, NTP hydrolyzing, and DNA or RNA unwinding [2, 87]. Some helicases also contain accessory domain(s) at the N- or C-terminus, such as nuclease domain and various protein-protein interaction domains. As shown in Figure 2, studies of human BLM helicase and its orthologs including budding yeast Sgs1 and *Drosophila* BLM revealed that its N-terminal region contains strand annealing activity [64]. Studies of the RecQ5*β* helicase revealed that the C-terminus is responsible for its annealing activity [55]. The annealing activity of PIF1 resides in its N-terminal domain [56]. Furthermore, studies of RECQ4 protein revealed that some missense mutants lose unwinding activity but still possess strand annealing activity [54]. These results suggest that the DNA unwinding and strand annealing activities can be uncoupled, but the question remains whether there is a conserved domain that controls annealing activity.

The Cortez lab found that only the second HP domain is required for the annealing activity of HARP [83] (Figure 2). Alignment of the HP domain with AH2 reveals a putative “HP-like” domain in the AH2 protein (residues 712–820) [82]. Although HP domain-like amino acids are found in the AH2, it is unlikely that the HP domain is a universal element that governs annealing activity across helicases. For example, the N-terminal region (residue 1–56) of RECQ1 [88] and the C-terminal region (aa 1072–1150) of the WRN helicase [52] are required for their respective annealing activities. Alignment of the “annealing domains” of these two RecQ helicases with the HP domains present in HARP and the HP-like domain in AH2 reveals no significant conserved residue. Although the N-terminal domain of RECQ4 and the C-terminal region of RECQ5 share certain identity and similarity, alignment of the N-terminus of RECQ4 and BLM, and the C-terminus of RECQ5 does not result in any significant homology (data not shown). Thus, it is unlikely that a single conserved domain is responsible for the annealing activity of these helicases. More conclusive data will be obtained when more annealing helicases are identified.

### 5.2. Protein Oligomerization Affects Helicase Rewinding Activity 

Certain helicases may self-assemble to form dimers or higher order oligomers, and this can influence their catalytic activity or biological function [1–3]. Thus, oligomerization might be important for the regulation of helicase annealing activity. Human RECQ1 helicase efficiently promotes strand annealing as a higher order oligomer (tetramer) while smaller oligomeric states (dimer or monomer) acting to separate duplex DNA [50, 88]. Electron microscopy reconstructions of the higher order oligomeric form revealed that a cage-like structure forms a hollow channel, which may facilitate the annealing activity of RECQ1 [50]. Both consistent and inconsistent with the findings of RECQ1, Hel112, a homologue of human RecQ5*β* in *Sulfolobus solfataricus*, exists as two predominant stable oligomeric states: monomer and dimer. Only the monomeric form has 3′-5′ DNA-helicase activity, while both the monomer and the dimer possess strand-annealing activity [76]. These findings raise the possibility that higher order oligomers promote annealing activity and smaller order oligomers promote unwinding activity. Nevertheless, additional studies are needed to address the relationship between the oligomerization and dual activities of helicases.

### 5.3. Does ATP Act as a Switch between the Unwinding and Rewinding Activity of Helicases?

For helicases that possess unwinding activity, it makes sense that ATP fuels the unwinding activity, in turn, inhibits the annealing activity. Indeed, this has been seen in DNA helicases such as BLM [53], Pif1 [56], as well as the *E. coli* Cas3 helicase [89]. It was also reported that, for the RNA helicase Ddx42p, ATP triggers RNA strand separation while ADP triggers annealing of complementary RNA strands [42]. However, for helicases with no detectable unwinding activity, it is largely unknown how ATP regulates their annealing activity. For example, how ATP inhibits CSB's annealing activity [58], while HARP and AH2 require ATP [14, 15].

Rather than fueling the unwinding activity by hydrolyzing ATP, ATP binding might cause a conformational change in the helicase that prevents annealing. For example, the strand-annealing activity of RECQ5*β* is strongly inhibited by ATP*γ*S, a poorly hydrolyzable analog of ATP. This effect is alleviated by mutations in the ATP-binding motif of RECQ5*β*, indicating that the ATP-bound form of the protein cannot promote strand annealing [55]. ATP*γ*S also inhibits the annealing activity of BLM [51], WRN [51], and CrhR helicases [47]. For RECQ1, ATP binding induces a conformational change in the protein that serves as a molecular switch from a strand-annealing to a DNA-unwinding mode [49]. If ATP indeed functions as a switch to regulate or balance the unwinding and rewinding activity of helicases, a promising avenue for future research will be to investigate the regulation mechanism, for example, structural determination of helicases with and without ATP. 

### 5.4. Other Proteins Regulate Helicase Annealing Activity 

RPA is a fundamental protein involved in all aspects of cellular metabolism (see review [90, 91]). In DNA repair processes, RPA physically coats ssDNA to protect it from degradation by nucleases and also serves as a scaffold protein to recruit other repair proteins (e.g., Rad51) to strand break sites. At the same time, RPA promotes checkpoint signaling after replication fork stalling through activation of ATR*·*ATRIP and RAD17 complexes. RPA has been shown to stimulate the unwinding activity of many helicases *in vitro*, including WRN [92, 93], BLM [94], RECQ1 [95], and FANCJ [96]. In turn, the strand annealing activity of RECQ1 [49], PIF1 [56], and CSB [58] is inhibited by RPA. In contrast, the unwinding activity of RECQ4 is inhibited by RPA [68]. RPA depletion causes a dramatic reduction in the formation of the annealing products in *Xenopus* egg extracts, suggesting that RPA is required for single-strand annealing [97]. The annealing helicase HARP associates with RPA, but whether it is just a physical interaction or if RPA modulates HARP's annealing function remains unknown. For AH2, yet to be identified proteins may regulate its nuclease and/or annealing activity.

In addition to RPA, several other proteins have been shown to promote annealing activity of helicases. The 65 kDa subunit of U2AF is reported to mediate the annealing of complementary single-stranded RNA or single-stranded DNA, which reverses the action of RNA helicase A [98]. The purified human p53 protein inhibits the unwinding activity of several DNA helicases, such as T antigen DNA helicase, DNA helicases I and II of *E. coli*, and RNA helicases p68, by promoting the rapid renaturation of complementary strands [37]. The endonuclease XPG is also reported to stimulate the annealing activity of the WRN helicase [99]. 

### 5.5. Post-Translational Modification of Helicases

Catalytic activities of proteins can be modulated by posttranslational modifications, such as phosphorylation/dephosphorylation, acetylation, ubiquitination, and SUMOylation. For example, endogenous RECQ1 is phosphorylated upon treatment of cells with ionizing radiation (IR), Ultraviolet (UV), and hydroxyurea (HU) [100]; BLM is phosphorylated by ATM in response to IR [101, 102], ATR in response to HU [103], and several other kinases–CK1 [104], cdc2 [105], and MPS1 [106]. WRN is phosphorylated by ATM and ATR in response to replication fork arrest [107, 108]. BLM is a SUMO substrate and SUMO modification regulates BLM's intranuclear localization [109]. WRN is acetylated by the acetyltransferase p300 [110]. HARP is phosphorylated by ATM, ATR, and DNA-PK in response to the DNA damage checkpoint [11, 81]. Indeed, the strand annealing activity of CSB is increased by dephosphorylation with phosphatase I and decreased by phosphorylation with CKII [58]. Thus, helicase function may in specific cases be regulated by post translational modification through modulation of its strand annealing activity. Nevertheless, it remains largely unknown how annealing activity is modulated by these protein modifications.

## 6. Cellular and Biological Significance of ****Helicase Annealing Activity 

The biological function of so-called real helicases, which possess unwinding activity that includes RecQ family helicases [4, 6], Pif1 [71], and Dna2 [72], has been extensively reviewed. The focus here is on annealing activity of helicases. The physiological relevance of helicase annealing activity is revealed by the finding that several mutations observed in SIOD patients result in defective annealing activity in HARP protein [14, 78, 111]. Although a limited number of annealing helicases have been identified, several biological functions have been indicated by related experimental evidence.

### 6.1. Stabilization of the Stalled Replication Fork

**Figure 3:**
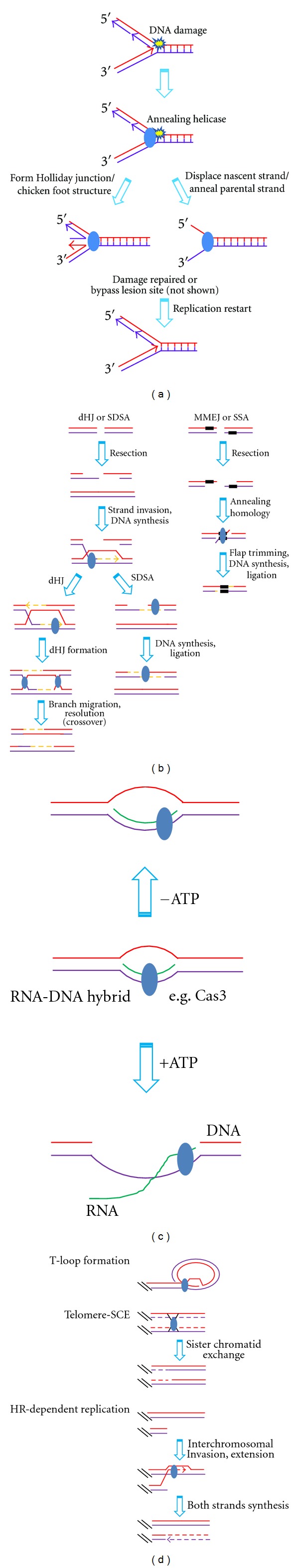
Purposed model of annealing helicase function in DNA replication, DNA repair, transcription, and telomere metabolism. (a) The annealing helicases function at stalled replication forks by catalyzing fork regression through Holliday junction migration (left) or displacing nascent strands and annealing parental strands (right). The replication fork proceeds until the DNA damage is repaired or bypassed. (b) Annealing helicases function in double-stranded DNA break repair. Annealing helicases might participate in homologous recombination repair through double Holliday junction (dHJ) or synthesis-dependent strand annealing (SDSA) pathways (left) or microhomology-mediated end joining (MMEJ) or single strand annealing (SSA) pathways (right). Noncrossover (uncommon) of dHJ is not shown. (c) Annealing helicases stabilize the DNA-RNA hybrid in transcription. Cas3 promotes RNA annealing to dsDNA without ATP and unwinding RNA from dsDNA with ATP. Model is adapted by analogy from one proposed for *E. coli* Cas3 helicase [89]. (d) Involvement of annealing helicases in telomere maintenance. Annealing helicases may catalyze the formation of T-loop structure or sister chromatid exchange and homologous recombination-dependent DNA replication in ALT cells. See text for details.

Stalled replication forks can arise during normal chromosome replication or in the presence of DNA lesions, but will collapse if being left unrepaired due to the presence of long stretches of ssDNA. Annealing helicases might stabilize stalled replication forks through pairing the parental ssDNA, migrating chicken foot/Holliday junctions structures, and/or directly participating in repair of the lesion (Figures 3(a) and 3(b)). Green fluorescent protein (GFP)-tagged HARP is recruited to stalled replication forks [11]. Compared with ssDNA and dsDNA, fork DNA is a preferred substrate for AH2 binding and ATPase activity [15], indicating that the forked DNA is a physical substrate for AH2. Because HARP and AH2 can act as an opposing force to unwinding activities *in vivo*, there are obvious potential implications for the role of HARP and AH2 in DNA replication and DNA repair activities (discussed below). HARP and AH2 might dictate their role in protecting stalled replication forks by minimizing the accumulation of ssDNA regions and facilitating the repair of collapsed replication forks during DNA replication [112]. Consistent with its function on ssDNA, four research groups have found that HARP associates with the RPA complex [11–13, 81], which possibly anchors HARP to the ssDNA. Cells depleted of HARP accumulate ssDNA and display increased sensitivity to aphidicolin and HU [12, 13]. DNA fibre analyses show that restart of replication forks after 2 hours of aphidicolin treatment is reduced in HARP-depleted cells [11, 81]. These data suggest that HARP promotes fork stability and restart by reannealing long stretches of ssDNA generated at stalled replication forks. Indeed, very recently, the Cortez lab demonstrated that *in vitro* HARP can bind and branch-migrate three-way and four-way DNA structures, and catalyze extensive fork regression of model replication forks [83]. 

The RecQ family helicases have been well recognized to function in damaged replication forks [113]. WRN- deficient cells are hypersensitive to replication blocking agents, including HU [107, 114] and DNA-interstrand cross-linking drugs [115]. BLM-deficient cells are also hypersensitive to HU [103] and Mitomycin C (MMC) [116]. Although less sensitive to HU as BLM-deficient and WRN- deficient cells, RECQ4-deficient cells are sensitive to HU [117]. RECQ1-depleted human cells are sensitive to HU [118] and camptothecin (CPT) [100]. The MEFs derived from Recq5-deficient mice are hypersensitive to CPT [119]. In addition, it has been reported that both BLM and WRN are recruited to blocked replication forks *in vivo* [120] and can catalyze fork regression *in vitro* [120, 121]. Moreover, the Orren lab recently demonstrated that WRN and BLM reestablish functional replication forks to overcome fork blockage [122]. All these evidence suggest that RecQ helicase, particularly BLM and WRN, participate in remodeling of stalled replication forks. However, it remains unknown how these helicases exert their annealing activity to contribute to replication fork restart.

In addition to the RecQ helicases, human Pif1 helicase specifically recognizes and unwinds DNA structures resembling putative stalled replication forks [66]. Using yeast ribosomal DNA as a DNA replication model, it has been shown that the events of replication fork block are increased in Dna2 mutants [123]. In particular, Dna2 is involved in Okazaki fragment processing [72], and it will be of interest to determine if its annealing activity stabilizes the lagging strand. Unlike HARP and AH2, it would seem most likely that proteins with both unwinding and annealing activities might coordinate those activities to catalyze strand exchange and/or branch migration, such as generating and migrating a Holliday junction or chicken foot structure for stalled DNA replication forks (Figure 3(a)).

### 6.2. DNA Repair

The function of the RecQ helicase family in DNA repair processes is well recognized [6]. DNA2 helicase/endonuclease is directly implicated in Rad51-dependent recombinational repair [124]. The CSB protein is known to function in transcription-coupled DNA repair (TCR) [73]. The Pif1 helicase plays critical roles in both nuclear and mitochondrial genome stability [71]. HARP is localized to DSB sites [80]. Cells with HARP depletion display a higher frequency of DSB [13, 81] and sensitivity to DNA-damage (e.g., IR, MMC, and CPT) [12]. HARP is phosphorylated by ATM, ATR, and DNA-PK *in vitro*, and the mobility of HARP in SDS gels is altered when it is isolated from cells treated with DNA damage (HU, IR, and UV radiation) [11]. Taken together, evidence suggested that annealing helicases are involved in DNA repair, but the question remains how the annealing activity contributes to DNA repair processes.

Of the various types of DNA damage, DSB may be the most common and cytotoxic to cells. DSBs are repaired by Non-Homologous End Joining (NHEJ), Homologous Recombination (HR), and Microhomology-Mediated End Joining (MMEJ) [125]. All three DNA repair pathways need pairing of complementary single-stranded DNA, particularly HR and MMEJ. There are mainly four forms of HR in higher eukaryotic cells: double Holliday junctions (dHJ) formed through crossover, synthesis-dependent strand annealing (SDSA) utilized by non-crossover, single-strand annealing (SSA) pathway between two repeat sequences, and break-induced replication (BIR) pathway that repairs “one-ended” DSBs. Both crossover and noncrossover HR pathways are initiated by Rad51 that searches and invades base-paired strands of homologous DNA molecules, then D-loop and HJs are formed consequently (Figure 3(b), left). RecQ helicases have strand exchange activity [51, 126] and HJs branch migration ability [120, 121]. In the dHJ pathway, it is very likely that the RecQ helicases, particularly WRN and BLM, coordinate their unwinding and annealing activities to separate intact double-stranded DNA and pair invading strand to template in the early stage, migrate HJs, and finally help to cleave HJs. SDSA is a mechanism in which homology-mediated repair of DSBs occurs without formation and resolution of ligated HJs; it anneals the newly synthesized strand with the single strand resulting from resection of the second end (Figure 3(b), left). MMEJ or SSA use microhomologous sequences (5–25 bp) or long homologies (>30 bp) to align broken ends before ligation (Figure 3(b), right) [127]. Nevertheless, dHJ, SDSA, MMEJ, and SSA pathways heavily rely on single-strand DNA-annealing activity driven by proteins. On the other hand, DNA helicases are recognized to play negative regulatory roles in recombination/repair through antirecombination, by disrupting presynaptic filaments prior to strand invasion, or by resolving D-loops before they can be extended and converted into replication forks. Although annealing helicases HARP and AH2 have not been investigated in the DNA repair pathways discussed above, both RECQ4 and BLM helicases are reported to be required to promote SDSA [128, 129]. In the MMEJ or SSA pathways, nuclease activity is required in the flap-trimming step (Figure 3(b), right). In fact, it has been reported that several helicases, including annealing activity containing helicase RECQ5*β* [130], BLM [131], and WRN [132], stimulate cleavage activity of Flap endonuclease 1 (FEN-1). 

### 6.3. Transcription

Besides their function in DNA replication and repair, another function of annealing helicases might be in DNA transcription (Figure 3(c)). Cas3 is a superfamily 2 helicase that possesses ATPase, helicase, and nuclease activities as evident in the Cas3 protein of *Streptococcus thermophilus *[133]. Recently, the *E. coli* Cas3 protein was found to promote R-loop (RNA : DNA hybrid) formation within duplex DNA in the absence of ATP and to disassemble R-loops in the presence of ATP [89], which is more relevant to structures formed in DNA transcription. It remains unknown whether such annealing helicase is present in human. Given the fact that the CSB protein is recruited to the site of a chromatin bound UV-stalled RNA polymerase II complex [134] and that the RECQ5 helicase physically and functionally associates with RNA polymerase II [135–138], it will be of interest to determine if the CSB and RECQ5 use their annealing activity to stabilize the transcription machine as a DNA damage response.

### 6.4. Telomere Metabolism

A number of RecQ helicases are implicated in telomere metabolism, including WRN [139], BLM [140], and RECQ4 [141]. Helicases Dna2 and Pif1 have also been implicated in chromosome end stability [142]. The purified recombinant PIF1 proteins bind telomeric DNA with a 100-fold higher affinity compared to random sequence, and telomere shortening was observed when PIF1 was overexpressed [143]. Telomere effects of Dna2 proteins have been reported in *S.  cerevisiae* and *S.   pombe* [144, 145]. It will be of interest to know how these helicases exert their strand annealing activity, in particular RECQ4 that has robust annealing activity, to function in telomere maintenance. Annealing helicases might participate in telomere metabolism, where single strand overhang could require an annealing helicase to form a more stable structure, such as T-loop (Figure 3(d)). For cells which operated by the Alternative Lengthening of Telomere (ALT) pathway, it is possible that strand annealing/strand exchange catalyzed by certain RecQ helicases may participate in telomere-sister chromatid exchange and HR-dependent DNA replication (Figure 3(d)). Coordination of strand annealing and unwinding activity at the G-rich telomeric end may influence telomere stability by affecting DNA replication and repair processes, such as resolving G4 DNA.

### 6.5. Chromatin Remodeling

HARP and AH2 belong to Swi2/Snf2 family that has been considered chromatin remodeling enzymes. The characteristic feature of this class of enzymes is that they contain a conserved ATPase domain with the seven classic helicase-related motifs. The Swi2/Snf2 ATPases are grouped within the SF2 helicase superfamily, and they have a distinct primary sequence signature defined by the spacing between helicase motifs III and IV as well as conserved features of their sequences within the domain [146]. Although most Swi2/Snf2 ATPases do not have duplex DNA strand separating activity, they retain other features of helicases, including directional DNA translocation fueled by ATP hydrolysis, participation in chromatin structure modeling, regulation transcription, and DNA repair.

Unlike HARP and AH2, not all helicase domains containing Snf2 family proteins have annealing activity, but like HARP and AH2, many of them have been identified to promote branch migration of HJs in an ATP hydrolysis-dependent manner. In prokaryotes, RuvAB and RecG have been shown to promote branch migration of HJs [147, 148]. In eukaryotes, several members of Snf2 can promote fork regression and/or branch migration of HJs, including Rad54 [149], Rad5 [150, 151], and HLTF [152]. CHD7, a helicase domain containing Snf2 protein accounted for the majority of CHARGE syndrome, plays a role in transcription regulation by chromatin remodeling [153]. Although FANCM is not a Snf2 family member, it consists of an N-terminal SF2 helicase domain and a C-terminal inactive endonuclease domain. FANCM is able to branch migrate HJs and replication forks *in vitro* [154, 155]. Very recently, several helicase domain containing Snf2 proteins, including SMARCA2 and SMARCA4, were identified to cause Nicolaides-Baraitser syndrome [156] and Coffin-Siris syndrome [157]. Interestingly, most of the patient mutations are located in the helicase domain, suggesting its importance for their function. However, it remains unknown whether they have strand annealing activity *in vitro*. In conclusion, it is a growing family of proteins that contain ATPase/helicase domain, which use their enzymatic activity to regulate chromatin structure and gene expression.

## 7. Conclusions and Perspectives

The discovery of annealing helicases establishes the presence of a class of enzymes that possess only rewinding activity and opens a new area of research. The range of proteins that function as annealing helicases remains to be determined. Researchers now hope to determine the biological function of HARP and AH2 more fully, as well as to discover more of these types of enzymes. Annealing helicases could also potentially be found for RNA-DNA and RNA-RNA hybrids, expanding the research into areas such as protein synthesis, RNA stability, and gene silencing. There is no doubt that more and more annealing helicases will be identified. The coordinated action of unwinding and annealing may play a role in fork regression or synthesis-dependent strand annealing, in the pathway for DSB repair, as well as in transcription and telomere metabolism. The challenge will then be to understand how cells regulate helicase unwinding and rewinding activity *in vivo*, and determine where or when the annealing activity of helicase is needed. From an experimental standpoint, it would be great interest to identify and characterize separation of function mutants which: (1) inactivate helicase activity but retain strand annealing; (2) inactivate strand annealing but retain helicase activity. Characterization of clinically relevant or engineered helicase core domain and auxiliary domain missense mutants may be valuable to determine which biological pathways/steps require strand annealing versus unwinding activity. Finally, a better understanding of the biological function of annealing helicases is likely to provide the basis for treating a variety of human disorders, such as SIOD of HARP, premature aging of RecQ helicases, and cancers.

## Figures and Tables

**Table 1:** Human helicases containing annealing activity.

Helicases	Unwinding activity (Polarity)	ATP requirement for annealing activity	Other activities	References
RNA helicase II (Gu, DDX21)	√	−		[40]
p68 family (p72, Ddx42p)	√	+ (Ddx42p); − (p68, p72)		[41, 42]
RNA helicase A (DHX9)	√ (3^′^-5^′^)	+		[44]
RECQ1	√ (3^′^-5^′^)	−		[49, 50]
WRN	√ (3^′^-5^′^)	−	3^′^ to 5^′^ exonuclease	[51, 52]
BLM	√ (3^′^-5^′^)	−		[51, 53]
RECQ4	√ (3^′^-5^′^)	−		[54]
RECQ5*β*	√ (3^′^-5^′^)	−		[55]
Pif1	√ (5^′^-3^′^)	−		[56]
Dna2	√ (5^′^-3^′^)	−	5^′^ to 3^′^ endonuclease; 3^′^ to 5^′^ exonuclease	[57]
CSB		−		[58]
HARP (SMARCAL1)		+		[11–14]
AH2		+		[15]
TWINKLE	√ (5^′^-3^′^)	−		[59]

Note: “+” stands for ATP is required; “−” for ATP not required.

## Abbreviations

ssDNA: Single-stranded DNA
ssRNA: Single-stranded RNA
dsDNA: Double-stranded DNA
HARP: HepA-related protein
SIOD: Schimke immunoosseous dysplasia
AH2: Annealing helicase 2
DSB: Double-strand break
NHEJ: Non-homologous end joining
HR: Homologous recombination
MMEJ: Microhomology-mediated end joining
SDSA: Synthesis-dependent strand annealing
SSA: Single strand annealing
HJ: Holliday junction
ALT: Alternative lengthening of telomere.
